# TNF−α Secreted from Macrophages Increases the Expression of Prometastatic Integrin αV in Gastric Cancer

**DOI:** 10.3390/ijms24010376

**Published:** 2022-12-26

**Authors:** Mi-Aie Hwang, Misun Won, Joo-Young Im, Mi-Jung Kang, Dae-Hyuk Kweon, Bo-Kyung Kim

**Affiliations:** 1 Personalized Genomic Medicine Research Center, KRIBB, Daejeon 34141, Republic of Korea; 2 Department of Integrative Biotechnology, College of Biotechnology and Bioengineering, Sungkyunkwan University, Suwon 16419, Republic of Korea; 3 KRIBB School of Bioscience, University of Science and Technology, Daejeon 34113, Republic of Korea; 4 R&D Center, oneCureGEN, Daejeon 34141, Republic of Korea

**Keywords:** gastric cancer, macrophage, TNF-α, integrin αV

## Abstract

The tumor microenvironment comprising blood vessels, fibroblasts, immune cells, and the extracellular matrix surrounding cancer cells, has recently been targeted for research in cancer therapy. We aimed to investigate the effect of macrophages on the invasive ability of gastric cancer cells, and studied their potential mechanism. In transcriptome analysis, integrin αV was identified as a gene increased in AGS cells cocultured with RAW264.7 cells. AGS cells cocultured with RAW264.7 cells displayed increased adhesion to the extracellular matrix and greater invasiveness compared with AGS cells cultured alone. This increased invasion of AGS cells cocultured with RAW264.7 cells was inhibited by integrin αV knockdown. In addition, the increase in integrin αV expression induced by tumor necrosis factor-α (TNF-α) or by coculture with RAW264.7 cells was inhibited by TNF receptor 1 (TNFR1) knockdown. The increase in integrin αV expression induced by TNF-α was inhibited by both Mitogen-activated protein kinase (MEK) inhibitor and VGLL1 S84 peptide treatment. Finally, transcription of integrin αV was shown to be regulated through the binding of VGLL1 and TEAD4 to the promoter of integrin αV. In conclusion, our study demonstrated that TNFR1–ERK–VGLL1 signaling activated by TNF-α secreted from RAW264.7 cells increased integrin αV expression, thereby increasing the adhesion and invasive ability of gastric cancer cells.

## 1. Introduction

Gastric cancer is known to be highly heterogeneous in both the intratumoral and intertumoral environments. One aspect of this heterogeneity occurs within the tumor microenvironment, which is highly permeated by cancer-related fibroblasts, immune cells, and stromal components [1]. One characteristic of the tumor microenvironment in gastric cancer is chronic inflammation derived from infection, such as *Helicobacter pylori*, which promotes cell survival and causes the upregulation of pathways that activate stem cell and epithelial cell proliferation [2]. *Helicobacter pylori* and other pathogens impair M1 macrophage responses, thereby leading to an M2-like state; these pathogens increase the rate of reactive oxygen species-induced macrophage apoptosis, which exacerbates the risk of disease progression [2]. Infection by *Helicobacter pylori* induces inflammation in the stomach microenvironment and is associated with the induction of cytokines, such as tumor necrosis factor-α (TNF-α), interleukin-1β, and interleukin-6, which lead to gastric cancer [3]. 

Macrophages are a major component of the tumor microenvironment and regulate various aspects of immunity. Depending on their activation status, macrophages can exert dual effects on tumorigenesis, either antagonizing the cytotoxic activity of immune cells or strengthening antitumor reaction [4]. Macrophages promote invasion and metastasis from the primary tumor sites through their ability to let cancer cells engage in an autocrine loop that promotes cancer cell migration [5]. Macrophage-derived cathepsins, SPARC, or CCL18 improve tumor cell adhesion to extracellular matrix proteins and promote tumor cell migration [5]. It has been reported that TNF-α, an inflammatory cytokine secreted from macrophages, promotes cancer cell proliferation, migration, and invasion in gastric cancer, colon cancer, breast cancer, and kidney cancer [3,6,7,8,9]. TNF is involved in various intracellular signaling mechanisms and has been reported to regulate the expression of the integrin family proteins in various diseases. TNF-α increased αVβ3 expression in human chondrosarcoma cells [10]. Fibroblast paracrine TNF-α increased integrin β5 expression in idiopathic pulmonary fibrosis [11]. TNF-α induced the activation of β-integrin function [12].

Integrins are a group of transmembrane proteins that act as cell–matrix adhesion receptors for transmitting signals and regulating various biological processes. To date, 18 α and 8 β subunits have been identified; these can directly form 24 known heterodimers and each α/β combination confers binding specificity of the extracellular domains to different ligands [13]. Many studies have also demonstrated the possibility of integrins as therapeutic targets in cancer treatment [14]. Integrins have been reported as diagnostic and prognostic markers for gastric cancer through various analyses of integrin expression and prognosis [15,16]. Integrin αV is a member of the integrin alpha chain family and can associate with up to five beta subunits to form αvβ1, αvβ3, αvβ5, αvβ6, and αvβ8 integrin receptors [17]. Integrin αV promotes the growth, migration, and invasion of gastric cancer cells [17]. Even through integrin αV is known as a target gene for FOSL1, TAZ, and SIN3B, there have been no reports of the mechanism of the regulation of integrin αV expression in gastric cancer [18,19,20].

In this study, we analyzed the pathways associated with the changes in gene expression induced by the coculture of macrophages cells and gastric cancer cells and identified integrin αV. In addition, the mechanism via which TNF-α-induced integrin αV promotes the metastasis of gastric cancer cells was verified.

## 2. Results

### 2.1. Integrin αV was Identified as a Gene with Increased Expression in AGS Cells Cocultured with RAW264.7 Cells

**Figure 1:**
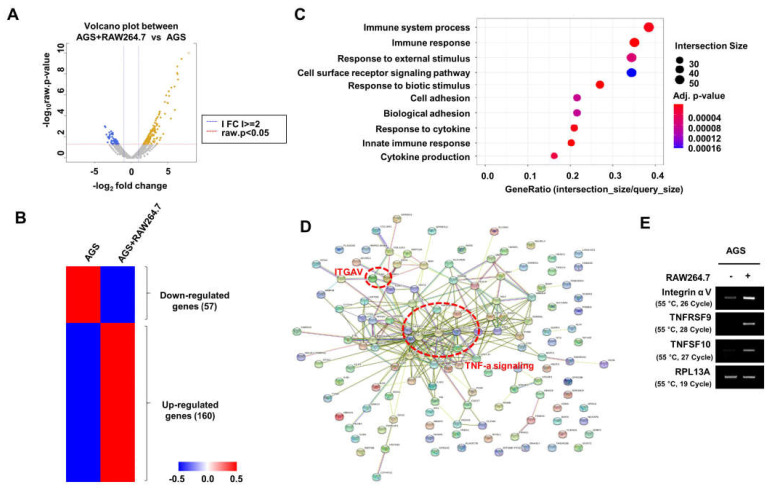
RNA−Seq analysis in AGS cells cocultured with RAW264.7 cells. (**A**,**B**) Volcano plot (**A**) and heat map (**B**) in human gastric cancer cells, AGS cells, and AGS cells cocultured with mouse macrophage RAW264.7 cells. (**C**) Gene ontology enrichment analysis. (**D**) STRING analysis. (**E**) The expression of integrin αV, TNFRSF9, and TNFSF10 was analyzed by RT−PCR (three replicates).

First, we performed RNA-Seq to confirm changes in the expression of genes in the relevant pathway in human gastric cancer cells (AGS cells) and AGS cells cocultured with macrophages (RAW264.7 cells) (Figure 1A). In the gene ontology enrichment analysis of the 160 genes that were increased in AGS cells cocultured with RAW264.7 cells, pathways related to immune response, cell adhesion, and cytokine response were enriched (Figure 1B). STRING analysis revealed the strong binding of TNF-α signaling-related genes (Figure 1C). We confirmed that the expression of TNFSF10, TNFRSF9, and integrin αV were top-ranked genes involved in TNF-α signaling (Figure 1D); among them, integrin αV, which was reported to play an important role in cell adhesion, was selected for further study.

### 2.2. Integrin αV Regulated the Adhesion and Invasion of AGS Cells Cocultured with RAW264.7 Cells

**Figure 2:**
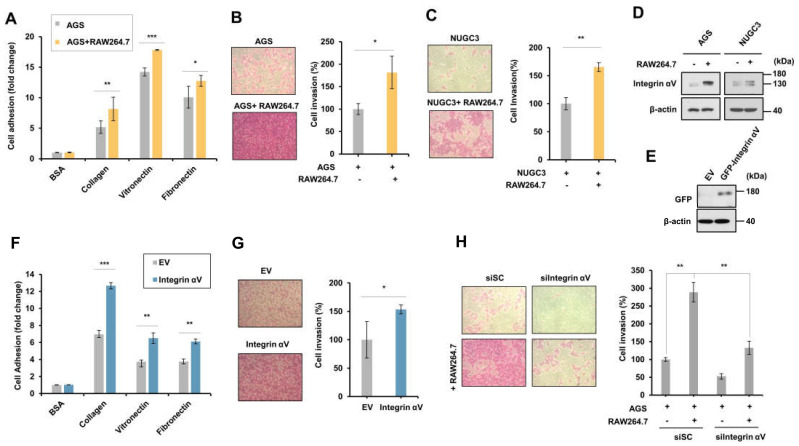
Integrin αV is involved in the invasion of AGS cells cocultured with RAW264.7 cells. (**A**) The effect of macrophages on the adhesion of gastric cancer cells. After coculture of AGS cells and RAW264.7 cells for 24 h, cell adhesion to the extracellular matrix (ECM)-coated plate was analyzed using sulforhodamine B (SRB) dye (*n* = 3). (**B**,**C**) The effect of macrophages on the invasion of gastric cancer cells. After coculture of gastric cancer cells and RAW264.7 cells for 24 h, cell invasion was analyzed by SRB assay (*n* = 3). (**D**) Integrin αV expression induced by coculture of RAW264.7 cells with AGS or NUGC3 cells. Integrin αV expression was analyzed by Western blotting (three replicates). (**E**) The expression of integrin αV in AGS cells transfected with pCMV3−HA (empty vector) or pCMV3−Integrin αV−GFP (two replicates). (**F**,**G**) The effect of integrin αV on adhesion (**F**) and invasion (G) of AGS cells (*n* = 3). (**H**) The knockdown of integrin αV inhibited the increase in the invasion of gastric cancer cells induced by macrophages. After AGS cells were transfected with siScramble (SC) or siIntegrin αV for 24 h, the cells were incubated with RAW264.7 cells for 24 h. Cell invasion was analyzed using the SRB assay (*n* = 3). Student’s *t*−test was used for statistical analyses. The bars indicate the S.D.; the asterisks denote significant differences (*** *p* < 0.001, ** *p* < 0.01, * *p* < 0.05) between the means of two groups.

Next, to verify the effect of macrophages on the adhesion and invasion of gastric cancer cells, the adhesion of AGS cells to the extracellular matrix (ECM) proteins, such as vitronectin, collagen, and fibronectin, was examined. AGS cells cocultured with RAW264.7 cells increased cell adhesion to ECM compared to the cells cultured with AGS alone (Figure 2A). The invasion of AGS and NUGC3 cells was also increased by coculture with RAW264.7 cells (Figure 2B,C). Furthermore, the protein expression of integrin αV was increased in AGS and NUGC3 cells by being cocultured with RAW264.7 cells (Figure 2D). Next, to determine the effect of integrin αV on the metastasis of gastric cancer cells, integrin αV was transfected into AGS cells (Figure 2E). Overexpression of integrin αV increased the adhesion and invasion of AGS cells (Figure 2F,G). By contrast, the invasion of AGS cells increased via coculture with RAW264.7 cells was also inhibited in AGS cells treated with siRNA of integrin αV or siIntegrin αV (Figure 2H). These results suggest that integrin αV is involved in the macrophage-induced invasion of gastric cancer cells.

### 2.3. Integrin αV Expression Was Increased in Gastric Cancer Cells through TNF-α/TNFR1/ERK Signaling

**Figure 3:**
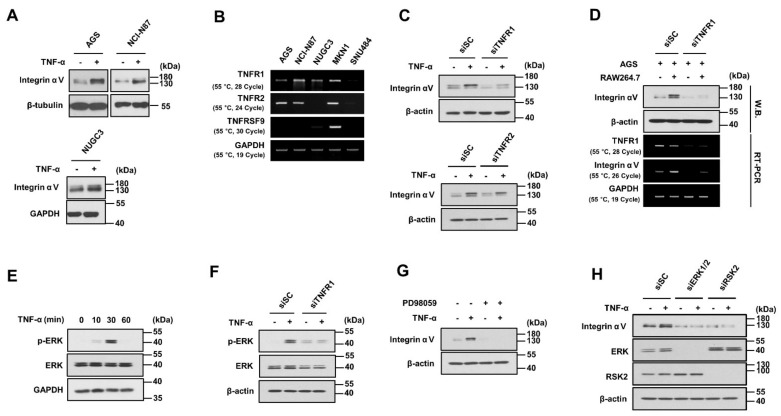
Increased integrin αV in gastric cancer cells through TNF−α/TNFR1/ERK signaling. (**A**) Increased integrin αV expression is induced by TNF−α in gastric cancer cell lines. Cells were treated with 20 ng/mL TNF−α for 24 h. Integrin αV expression was analyzed by Western blotting (three replicates). (**B**) The expression of TNFRSF9, TNFR1, and TNFR2 in gastric cancer cell lines. TNFRSF9, TNFR1, and TNFR2 expression was analyzed by RT−PCR (two replicates). (**C**) The effect of TNFR1 and TNFR2 knockdown on TNF−α−induced expression of integrin αV. AGS cells were transfected with 40 nM siScrambled or siRNA targeting TNFR1 or TNFR2 for 24 h and then treated with TNF−α for 24 h (three replicates). (**D**) The effect of TNFR1 on the expression of integrin αV increased by the coculture of AGS cells with RAW264.7 cells. The mRNA and protein expression of integrin αV was analyzed by Western blotting (three replicates) or RT−PCR (four replicates). (**E**) Increased phosphorylation of ERK by TNF−α. AGS cells were incubated with serum−free medium for 24 h and then treated with TNF−α at the indicated times. Phospho−ERK and ERK expression was analyzed by Western blotting (three replicates). (**F**) The effect of TNFR1 on the TNF−α−induced phosphorylation of ERK. AGS cells transfected with siScramble or siTNFR1 were treated with TNF−α for 30 min (four replicates). (**G**) The effect of ERK on TNF−α−induced integrin αV expression. After pretreatment with PD98059 for 1 h, AGS cells were treated with TNF−α for 24 h (three replicates). (**H**) The effect of ERK and RSK2 on the TNF−α−induced expression of integrin αV. AGS cells were transfected with siScramble, siERK1/2, or siRSK2 for 24 h and then the cells were treated with TNF−α for 24 h. The protein expression was analyzed by Western blotting (four replicates).

The effect of TNF-α, a cytokine secreted from macrophages, on integrin αV expression was investigated. TNF-α increased integrin αV expression in AGS, NCI-N87, and NUGC3 human gastric cancer cells (Figure 3A). Subsequently, RT-PCR was performed to examine the expression of TNF receptors in gastric cancer cell lines. The expression of TNF receptor 1 (TNFR1) and TNF receptor 2 (TNFR2) was increased in AGS, NCI-N87, NUGC3, and MKN1 cells; meanwhile, the receptor TNFRSF9 was expressed only in MKN1 cells (Figure 3B). This increase in integrin αV expression induced by TNF-α was inhibited by treatment with siTNFR1 but not siTNFR2 (Figure 3C). Furthermore, TNFR1 knockdown inhibited the increased integrin αV expression induced by coculturing with RAW264.7 cells (Figure 3D). TNF-α induced phosphorylation of ERK in AGS cells (Figure 3E), however this was inhibited by TNFR1 knockdown (Figure 3F). Pretreatment with PD98059, a MEK inhibitor, reduced this increase in integrin αV expression induced by TNF-α (Figure 3G). Furthermore, the knockdown of RSK2, a downstream target of ERK, inhibited the increase in integrin αV expression induced by TNF-α (Figure 3H). These results suggest that TNF-α activates TNFR1 and then phosphorylates ERK, thereby regulating integrin αV expression.

### 2.4. VGLL1 Regulates the Invasion of Gastric Cancer Cells through Integrin αV

**Figure 4:**
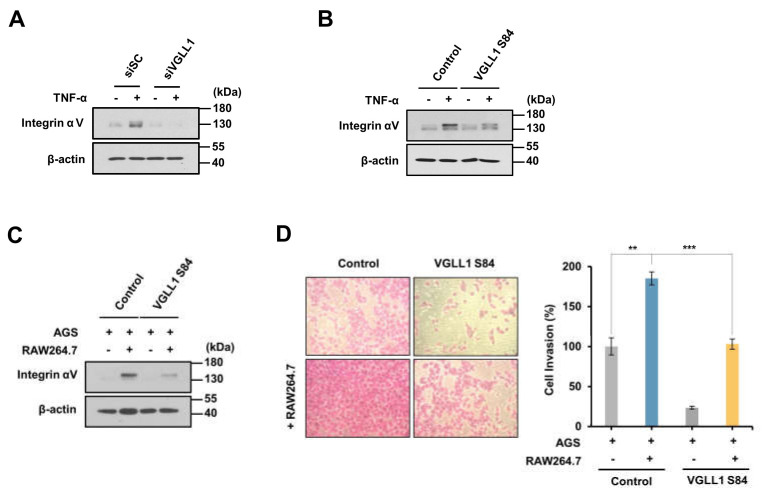
VGLL1 is involved in TNF−α−induced expression of integrin αV. (**A**) The effect of VGLL1 on TNF−α−induced integrin αV expression. AGS cells were transfected with siScramble or siVGLL1 for 24 h and then the cells were treated with TNF−α for 24 h. Integrin αV expression was analyzed by Western blotting (four replicates). (**B**) The effect of VGLL1 S84 peptide on TNF−α−induced expression of integrin αV. After incubation with 20 µM control peptide or VGLL1 S84 peptide for 1 h, AGS cells were treated with TNF−α for 24 h. Integrin αV expression was analyzed by Western blotting (three replicates). (**C**,**D**) AGS cells were cocultured with RAW264.7 cells for 24 h in the presence of control or VGLL1 S84 peptide. (**C**) The effect of VGLL1 on integrin αV expression in AGS cells cocultured with RAW264.7 cells. Integrin αV expression was analyzed by Western blotting (three replicates). (**D**) The effect of VGLL1 on the invasion of AGS cells cocultured with RAW264.7 cells. Cell invasion was analyzed using the SRB assay (*n* = 3). ** *p* < 0.01, *** *p* < 0.001.

We next investigated the effect of VGLL1, which is known as a downstream protein of RSK2, on the increase in integrin αV expression by TNF-α. The TNF-α-induced expression of integrin αV was shown to be suppressed in AGS cells treated with siVGLL1 compared with cells treated with siScramble (Figure 4A). In addition, the expression of integrin αV was shown to be suppressed even in the presence of TNF-α by the treatment with VGLL1 S84 peptide, which inhibits VGLL1 phosphorylation (Figure 4B). In addition, the expression of integrin αV increased by coculturing of AGS cells and RAW264.7 cells was inhibited by VGLL1 S84 peptide (Figure 4C). Finally, we observed that the VGLL1 S84 peptide inhibited the invasion of gastric cancer cells increased by coculture with RAW264.7 cells (Figure 4D). These results suggest that TNF-α in macrophages induces the phosphorylation of VGLL1, which regulates the invasion of gastric cancer cells.

### 2.5. Transcription of Integrin αV Is Regulated by VGLL1 and TEAD4

**Figure 5:**
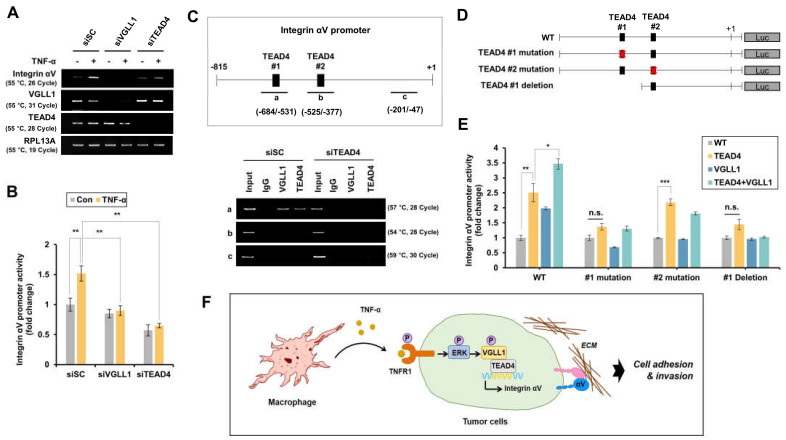
TNF−α−induced integrin αV transcription is regulated through TEAD4. (**A**) AGS cells were treated with siScramble, siVGLL1, or siTEAD4, and then treated with TNF−α for 24 h. The mRNA expression of VGLL1, TEAD4, integrin αV, and RPL13A was analyzed by RT−PCR (three replicates). (**B**) The effect of VGLL1 and TEAD4 on integrin αV promoter activity. AGS cells were transfected with integrin αV−Luc and Renilla−Luc for 24 h and then cells were treated with siScramble, siVGLL1, or siTEAD4 for 24 h. After serum starvation for 24 h, the cells were treated with TNF−α for 24 h. The promoter activity of integrin αV was normalized by Renilla−Luc (*n* = 3). (**C**) In AGS cells treated with siScramble or siTEAD4, the TEAD4 binding site in the integrin αV promoter was analyzed by ChIP assay (two replicates). (**D**) The construction of various luciferase reporters under the control of the integrin αV promoter. (**E**) Integrin αV promoter activity containing modified TEAD4−binding sites was measured in AGS cells (*n* = 3). (**F**) Model of TNF−α−induced integrin αV expression. Student’s *t*−tests was used for statistical analyses. The bars indicate the S.D.; the asterisks denote significant differences (*** *p* < 0.001, ** *p* < 0.01, * *p* < 0.05) between the means of two groups.

VGLL1 is known to bind to the transcription factor TEAD4 to regulate the expression of target genes. Therefore, it was examined whether TEAD4 regulates the transcription of integrin αV. The increased mRNA expression and promoter activity of integrin αV by TNF-α were shown to be inhibited by siTEAD4 (Figure 5A,B). For the binding of TEAD4 in the promoter region of integin αV, two TEAD4 binding sites (−577/−567, −513/−503) were previously identified [19] and Chromatin Immunoprecipitation (Chip) assays indicate that TEAD4 and VGLL1 bind to the first TEAD4 binding site of the promoter of integrin αV (Figure 5C). Furthermore, when an integrin αV promoter with mutated or deleted TEAD4 binding site was constructed (Figure 5D) and its binding activity was tested, the increase in integrin αV promoter activity by VGLL1-TEAD4 binding was suppressed when the first TEAD4 binding site was mutated or deleted but remained unaffected when the second TEAD4 binding site was mutated (Figure 5E). These results suggest that the TNF-α-induced integrin αV expression is regulated through VGLL1-TEAD4 binding.

## 3. Discussion

In this study, we have verified that TNF-α secreted from macrophages promotes cancer metastasis by increasing integrin αV expression in gastric cancer cells. When AGS cells were cocultured with RAW264.7 cells, the cell adhesion and the invasion of AGS cells increased; moreover, the expression of TNF-α-related genes was also increased. In AGS cells, the expression of integrin αV was increased by treatment with TNF-α or co-culturing with RAW264.7 cells. In addition, TNFR1 knockdown in AGS cells also inhibited TNF-α-induced integrin αV expression. This supports the existing view that integrin αV is important for the proliferation and metastasis of gastric cancer.

The importance of integrin as a target for cancer treatment has been well recognized for several years, and numerous integrin inhibitors have been developed [21,22]. Various integrin inhibitors are being tested in clinical trials, but successful results have not been obtained. As an intracellular signal transduction mechanism, ECM-ligated integrins initiate prosurvival signaling by BCL-2, FLIP factor-kB, NF-kB, and PI3K-AKT. In an unligated state, the integrins initiate a process known as integrin-mediated death via the cleavage of caspase-8 [21]. Although the regulatory mechanism of integrin in cancer cells has been reported, studies on the mechanism through which integrin expression is regulated by the tumor microenvironment remain uncommon. We demonstrated the regulation of integrin αV expression through the TNF-α–ERK–VGLL1–TEAD4 pathway in the coculture of gastric cancer cells with macrophages cells.

In liver cancer cells, integrin αV dose not bind to YAP, but instead binds to the promoter together with TEAD4 as a target gene for TAZ [19]. In gastric cancer, we confirmed that VGLL1, which has a similar structure to TAZ, binds with TEAD4 to the integrin αV promoter in the presence of TNF-α. The binding site of VGLL1 was identified by mutating or deleting the TEAD4 binding sites in the integrin αV promoter. Furthermore, it was confirmed that the correlation between VGLL1 and integrin αV expression in gastric cancer patients was significant (Supplementary Figure S1). This suggests that the expression of integrin αV can be regulated by PI3K [23], TGF-β [24], TNF-α, which are upstream signaling proteins of VGLL1, in addition to the YAP/TAZ signaling.

The function of TNF in immune cells is generally well established, and studies related to cancer malignancy have also been reported. The mRNA and protein expression of TNF have been detected in malignant and stromal cells from human cancer biopsy samples [25,26,27]; moreover, plasma TNF levels have been found to be increased in some patients with cancer, notably those with poor prognosis [28,29]. TNF in the tumor microenvironment can induce cancer malignancy through genetic damage or directly affect the EMT [25,30]. In human bile duct sarcoma cells, TNF induced DNA and RNA editing enzymes, activation-induced cytidine deaminase, which led to mutations in genes such as TP53 and MYC [31]. TNF antagonists inhibit cytokine and chemokine production, inflammatory cell recruitment, angiogenesis, and extracellular substrate degradation [25,32]. Cancer metastasis suppression experiments using anti-TNF antibodies have been verified by single or combined treatment in various cancer types since they were first reported in 1993 [25]. A Phase 1 study of the anti-TNF antibody infliximab reported disease stabilization in 7 out of 41 patients with progressive cancer [33].

Recently, studies have reported a tumor microenvironment promoting tumor malignancy in several carcinomas. Integrin α11-expressing cancer-associated fibroblast induces breast cancer metastasis via the platelet-derived growth factor receptors (PDGFRs)–JNK pathway [34]. In the analysis of transcriptome data from hypoxic and normoxic cultured human peripheral blood mononuclear cell (PBMC)-derived macrophages, it was found that C-X-C motif chemokine ligand 8 (CXCL8) expression was increased, and CXCL8 is reportedly involved in gastric cancer malignancy [35]. The increased expression of ECM metalloproteinase inducer (EMMPRIN, CD147) induced by the coculture of human renal carcinoma A498 cells and human breast carcinoma MCF-7 cells with human monocyte-like U937 cells promotes angiogenesis [36]. When human lung cancer A549 cells and RAW264.7 cells were cocultured, the expression of metastasis-related genes, such as MMPs and VEGF, was increased [37,38].

We confirmed that immune system processes, immune response pathways, and cell adhesion pathways were involved through the coculture of macrophages and gastric cancer cells. However, clinical research and development of therapeutic agents that inhibit substances secreted by macrophages have not yet been explored for patients with gastric cancer. Furthermore, it is necessary to study immunotherapeutic agents that can increase the efficacy of the gastric cancer drugs currently used in clinical practice. We were interested in ascertaining whether the suppression of TNFR in gastric cancer cells could increase the efficacy of anticancer drugs, and it was confirmed that oxaliplatin and cisplatin displayed a greater increase of the inhibitory efficacy of cell proliferation in the siTNFR1-treated group compared with the control group (Supplementary Figure S2). Our study confirmed that the secretion of TNF-α by macrophages promotes the metastasis of gastric cancer cells, thereby providing a basis for the use of TNF/TNFR inhibitors for the development of gastric cancer treatment agents. Consequently, experiments to confirm the effects of the coadministration of anticancer drugs and TNF inhibitors in animal models are required.

## 4. Materials and Methods

### 4.1. Cell Lines

Human gastric cancer cells (AGS, NCI-N87, and NUGC3) were purchased from the Korea Cell Line Bank (Seoul, Korea) and cultured in Roswell Park Memorial Institute -1640 medium containing 10% fetal bovine serum (FBS) and 1% penicillin/streptomycin. RAW264.7 murine macrophages cells were provided by Dr. Haiyoung Jung (KRIBB, Korea) and cultured in Dulbecco’s modified Eagle medium containing 10% FBS and 1% penicillin/streptomycin. All cells were maintained in an incubator at 37 °C with 5% CO_2_.

### 4.2. Reagents

TNF-α was purchased from R&D Systems (Minneapolis, MN, USA). Vitronectin was purchased from Gibco (Frederick, MD, USA). Collagen and PD98059 were purchased from Sigma (St. Louis, MO, USA). Bovine serum albumin (BSA) was purchased from RMBio (Missoula, MT, USA). Fibronectin was purchased from BD Biosciences (Franklin Lakes, NJ, USA).

### 4.3. Plasmids and siRNAs

pCMV3-Integrin αV-GFP was purchased from Sino Biological (Beijing, China). Fragments of the integrin αV promoter (−815/+174) was amplified by PCR using HEK293T genomic DNA as a template. The primers used for PCR amplification were 5′ CGAGTGTAATTAACGACCATTAATTAAC 3′ (forward) and 5′ GGTCCACAAACACTGAACTTAATTACGG 3′ (reverse). The amplified DNA fragment was inserted into the EcoRV and BglII site of pGL4.17-Luc2/neo. The integrin αV promoter, which contained mutation or deletion in the TEAD4 binding site, was generated using EZchange™ Site-directed Mutagenesis Kit (Enzynomics, Daejeon, Korea). The sequences of primer pair were used as follows: TEAD4 #1 mutation (5′-CAACACTTGCAAGAGGCTATGCTGGCTTTC-3′ and 5′-CCCGTAAAGCTTAACTTAGTAGTCAAAG-3′), TEAD4 #2 mutation (5′-GAGTGTAATTAACGACCATTAATTAAC-3′ and 5′-GGTCCACAAACACTGAACTTAATTACGG-3′), and TEAD4 #1 deletion (5′-GATATCGTGTTTGTGGAATGGAGTG-3′ and 5′-AGATCTGCCGCTCTCTTCTCGGCT-3′).

siRNA targeting integrin αV(3685-1), TNFR1(7132-1), and TNFR2 (7133-1) were purchased from Bioneer (Daejeon, Korea). The sequences of scrambled control siRNA and siRNA targeting VGLL1, TEAD4, ERK1, ERK2, and RSK2 have been reported previously [24].

### 4.4. Cocultures

RAW264.7 macrophages were cultured separately with gastric cancer cells in a 0.4 μm Transwell system (SPL, Pocheon, Gyeonggi-do, Korea). AGS cells (3 × 10^5^ cells) were seeded in six-well plates in serum-free medium. After 24 h, RAW264.7 cells (2 × 10^5^) cells were seeded in Transwell inserts in serum-free medium.

### 4.5. Transcriptome Analysis

The total RNA extracted from AGS cells and AGS cells cocultured with RAW264.7 cells was constructed into a library using TruSeq Stranded mRNA LT sample prep kit. Sequencing was performed using the Illumina platform (Macrogen, Seoul, Korea). The dataset is available in GEO DataSet of the NCBI Database under the accession number GSE220531.

### 4.6. Reverse Transcription–Polymerase Chain Reaction

AGS cells (3 × 10^5^ cells) were seeded in six-well plates. RNA was extracted from cells and isolated using Trizol reagent (Invitrogen, Carlsbad, CA, USA) in accordance with the Trizol protocol and cDNA was synthesized using TOP-script RT DryMIX (Enzynomics). RT-PCR was performed using 2× Hot TaqMasterMix (MGmed, Seoul, Korea). Primer pairs for integrin αV (P254519V), TNFRSF9 (P201215V), TNFSF10 (P144740V), TNFR1 (P253379V), TNFR2 (P199275V), VGLL1 (P227101V), TEAD4 (P130115V), and GAPDH (P267613V) were purchased from Bioneer. The sequences of RPL13A primer pair were as follows: (F) 5′-CATCGTGGCTAAACAGGTAC-3′, (R) 5′-GCACGACCTTGAGGGCAGC-3′.

### 4.7. Western Blotting

Cells were lysed in RIPA buffer (Millipore, Billerica, MA, USA) containing protease inhibitor cocktail (Roche, Basel, Switzerland). The lysates were quantified using a protein assay kit (Bio-Rad, Hercules, CA, USA) and the obtained proteins (20 μg) were separated by SDS-PAGE and transferred to a polyvinylidene difluoride membrane. The proteins were identified using the following antibodies: anti-VGLL1 [2000:1, 10124-2-AP; Proteintech (Rosemont, IL, USA)]; anti-TEAD4 [2000:1, ab58310; Abcam (Cambridge, MA, USA)]; anti-integrin αV (1000:1, 4711S), anti-β-tubulin (3000:1, 2128S), anti-p-ERK (3000:1, 4370S), anti-ERK (3000:1, 4695S), and anti-RSK2 (2000:1, 5528S) (Cell Signaling Technology, Danvers, MA, USA); anti-GAPDH [5000:1, LF-PA0212; AB FRONTIER (Seoul, Korea)]; and anti-β-actin [3000:1, SC-47778; Santa Cruz Biotechnology (Dallas, TX, USA)]; anti-GFP [2000:1, MA5-15256; Thermo Fisher (Waltham, MA, USA)].

### 4.8. Cell Adhesion and Invasion Assay

For the adhesion assay, cells were seeded in 96-well plates coated with BSA (1%), vitronectin (10 μg/mL), fibronectin (10 μg/mL), and collagen (50 μg/mL). The cells were allowed to attach for 30 min and nonadherent cells were removed by washing with 0.1% BSA. For the invasion assays, we used chambers with a 0.8 μm pore membrane in 24-well inserts (BD Biosciences). We seeded the cells into the upper part of each chamber, which was coated with Matrigel (BD Biosciences), and the lower compartments were filled with the medium. To analyze the adherent or invasive cells, 0.4% sulforhodamine B (SRB) was used to stain the cells after fixation with 10% formalin, as described previously [39]. Unbound dye was washed with 0.1% acetic acid; protein-bound dye was dissolved in 10 mM Tris, and its optical density was measured at 540 nm.

### 4.9. Luciferase Assay

The activity of the integrin αV promoter was determined by the dual-luciferase reporter system (Promega, Madison, WI, USA). Cells were transfected with pGL4.17-Integrin αV promoter-luc and pRL-SV40 encoding firefly luciferase (Renilla-Luc) using Turbofect Transfection Reagent (Thermo Fisher). Cells were harvested 48 h after transfection and lysed in 1× passive lysis buffer (Promega). Luciferase activity was measures using a multi-mode microplate reader (SYNERGY/HTX, BioTek, Winooski, VT, USA) and then normalized to the activity of Renilla luciferase.

### 4.10. Chromatin Immunoprecipitation (ChIP) Assay

ChIP assays were performed using EZ-Chip^TM^ Chromatin Immunoprecipitaion kit (Merck Millipore, Buulington, MA, USA) as previously described [23]. Chromatin was sheared by sonication on ice. Chromatin lysates were incubated with antibodies targeted against VGLL1, TEAD4, or with normal mouse IgG overnight at 4 °C, and the protein was then digested using proteinase K. ChIP-enriched DNA was PCR-amplified using three primers: integrin αV-a (5′-CATACAACCGCAGCTAACAAACTGG-3′ and 5′-CAGAAAGCCAGCATAGCCTCTTGC-3′), integrin αV-b (5-CCGTAATTAAGTTCAGTGTTTGTGG-3′ and 5′-CCGTGCGACTGTTGTTTAAAAATG-3′), and integrin αV-c (5′-GTACGCTGAGCTCTCCCCTGTAGAAG-3′ and 5′-GAGGAGACCTGGAAGGAGGAGGAG-3′).

## 5. Conclusions

The coculture of RAW264.7 cells and AGS cells increased the expression of integrin αV through the TNFR–ERK–VGLL1 pathway; this subsequently increased the adhesion and invasion of gastric cancer cells. Moreover, this effect was also observed after treatment with TNF-α; therefore, we suggest that TNF-α secreted by macrophages plays an important role in the metastasis of gastric cancer cells.

## Data Availability

The dataset is available in the NCBI Database of GEO datasets under the data series accession number GSE220531.

## Supplementary Materials

The following supporting information can be downloaded at: https://www.mdpi.com/article/10.3390/ijms24010376/s1.
