# Supplementary material for: TNF−α Secreted from Macrophages Increases the Expression of Prometastatic Integrin αV in Gastric Cancer
Source: Int J Mol Sci. 2022 Dec 26;24(1):376. doi: 10.3390/ijms24010376 (PMC9820470; doi:10.3390/ijms24010376)
Supplement: Supplementary file 1 [file ijms-24-00376-s001.zip › ijms-2041683-supplementary.pdf]

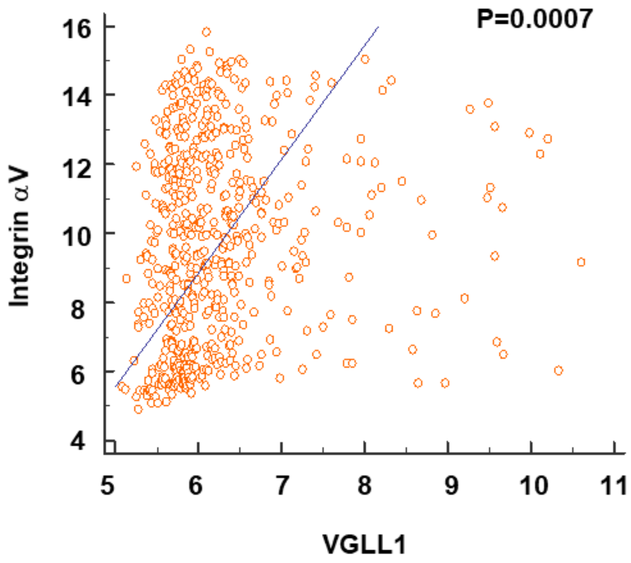

Supplementary Figure S1.

The expression correlation of VGLL1 with integrin aV in the gastric cancer cohort data (Severance Hospital (Seoul, South Korea), n = 565). Microarray data were available at the National Center for Biotechnology Information Database of GEO datasets under the data series accession numbers GSE13861 and GSE84437.

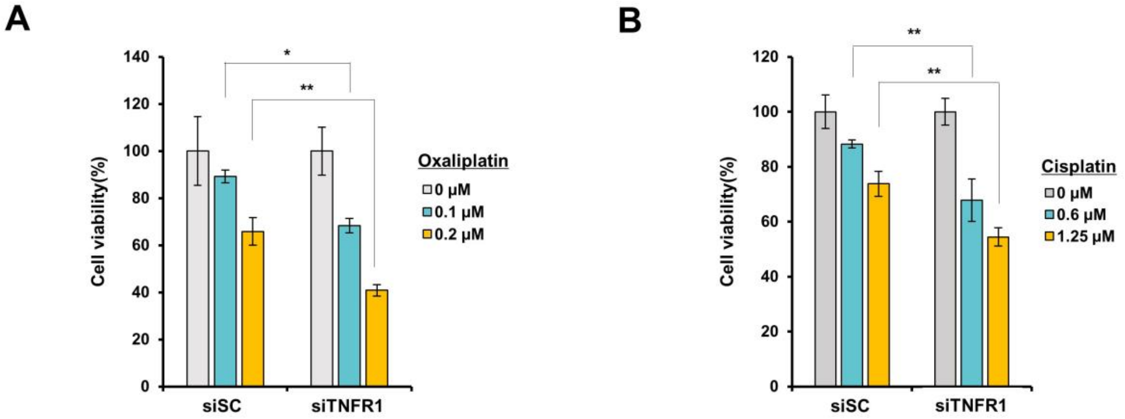

Supplementary Figure S2.

Inhibition of TNFR expression increases the inhibitory effect of anticancer drugs on gastric cancer cell growth. AGS cells were treated with siScramble or siTNFR1, and then treated with oxaliplatin (A) or cisplatin (B) for 72 h. Cell viability was analyzed using the SRB assay ($n = 3$). *** $p < 0.001$, ** $p < 0.01$, * $p < 0.05$.
